# The implications of magnetic resonance angiography artifacts caused by different types of intracranial flow diverters

**DOI:** 10.1186/s12968-021-00753-0

**Published:** 2021-06-07

**Authors:** Batur Halitcan, Sayin Bige, Balci Sinan, Akmangit Ilkay, Daglioglu Ergun, Alagoz Fatih, Arat Anil

**Affiliations:** 1 Department of Radiology, Ministry of Health Ankara City Hospital, Ankara, Turkey; 2 Department of Radiology, Hacettepe University Hospitals, Ankara, Turkey; 3 Department of Neurosurgery, Ministry of Health Ankara City Hospital, Ankara, Turkey

**Keywords:** Flow diverter, Aneurysm, Angiography, Time of Flight, Artifact

## Abstract

**Background:**

Serial cerebral angiographic imaging is necessary to ensure cerebral aneurysm occlusion after flow diverter placement. Time-of-flight (TOF)-magnetic resonance angiography (MRA) is used for this purpose due to its lack of radiation, contrast media and complications. The comparative diagnostic yield of TOF-MRA for different flow diverters has not been previously analyzed.

**Purpose:**

To evaluate the diagnostic accuracy of TOF-MRA in cerebral aneurysms treated w divertersith different flow diverters.

**Materials and Methods:**

Flow-diverted patients whose cerebral follow-up MRA and digital subtraction angiograms (DSA) were obtained within 6 weeks were retrospectively identified. The DSA (as gold standard) and MRA images of these patients were compared by two readers (blinded to both patient data and endovascular procedure data) for residual aneurysms and the status of the parent artery for each type of flow diverter. In a second group of patients, magnetic susceptibility artifacts were manually measured and compared for different FDs.

**Results:**

Seventy-six patients (85 aneurysms) were included in group one, and 86 patients (95 aneurysms) were included in group 2. TOF-MRA and DSA showed almost perfect agreement for residual aneurysms (κ = 0.88, *p* < 0.001) (positive predictive value (PPV) = 1.00, specificity = 1.00, negative predictive value (NPV) = 0.89, sensitivity = 0.89). Intermodality agreement (κ = 0.97 vs. κ = 0.74, *p* < 0.005) and sensitivity (0.97 vs. 0.77, NPV: 0.96 vs. 0.77) were highest with nitinol stents. MRA and DSA showed no agreement for occluded or stenotic parent vessels (κ = 0.13, *p* = 0.015, specificity = 0.44, NPV = 1.00, sensitivity = 1.00). Specificity was lower in chromium-cobalt based FDs than in nitinol devices (specificity = 0.08 vs. 0.60). Chromium-cobalt stents generated the largest artifacts (*p* < 0.005). The size of the device-related artifact, in millimeters, increased in respective order, for the Silk, Derivo, Pipeline and Surpass devices.

**Conclusion:**

Unlike DSA, TOF-MRA is susceptible to dissimilarities between flow diverters. MRA is not well-suited for research studies comparing different flow diverters. Nitinol FDs appear to be advantageous for TOF-MRA follow-up so as not to miss small aneurysm remnants or clinically relevant parent artery stenosis.

## Introduction

Aneurysm obliteration after flow diversion occurs over months. Serial invasive angiograms are not suitable to monitor this evolution. Consequently, noninvasive imaging is of paramount importance for the evaluation of flow-diverted aneurysms. Although some data exist in the literature regarding the magnetic resonance angiography (MRA) assessment of aneurysms after flow diverter placement, a comparative evaluation of MRA for different flow diverters has not been previously reported. Based on in vitro data [1] and on our clinical observations, we speculated that the choice of flow diverters may alter the MRA findings in the clinical setting. Since the unique physical and mechanical components of various flow diverters can potentially alter MRA images, we wanted to evaluate the diagnostic accuracy of time-of-flight(TOF)-MRA in cerebral aneurysms treated with different types of flow diverters.

## Materials and methods

### Study population

Records of all patients we treated with single-layer FDs (Tables 1 and 2) were retrospectively retrieved from the hospital information system. In the first group of patients, those with follow-up MRA and digital subtraction angiography (DSA) performed within 6 weeks (42 days) were identified. Patients with coiled aneurysms, multiple flow diverters that overlapped with each other, stents (previously placed or placed during the same treatment session, in the vicinity of the flow diverter) or previously clipped aneurysms were excluded due to susceptibility artifacts that can potentially interfere with the assessment of either the aneurysm sac or the parent artery. Since the majority of the MRA follow-ups were obtained with 1.5 T scanners, those patients who had a follow-up MRA with 3 T scanners were also excluded as field strength is known to influence artifact size (2). If, immediately after the procedure, there was computed tomography (CT)/CT angiography (CTA) or MRA evidence of substantial acute postprocedure thrombosis within the aneurysm, the aneurysm was not included for further analysis. Patients were not excluded if the DSA or MRA were performed for the treatment/evaluation of another aneurysm in a different arterial territory or if the patient had more than 1 flow-diverted aneurysm in different arterial territories (other hemisphere or posterior circulation). The DSA and MRA images of these patients were evaluated.

**Table 1:** Demographic properties of study population

Number of patients	111
Female	70 (63%)
Age(mean ± SD)	55 ± 16
Number of aneurysms	121
Anatomical distribution of aneurysms	
Left ICA	50 (41.3%)
Right ICA	44 (36.4%)
Left MCA	4 (3.3%)
Right MCA	13 (10.7%)
Left ACA	3 (2.5%)
Right ACA	2 (1.7%)
Left PICA	1 (0.8%)
Left VA	1 (0.8%)
Right PCA	1 (0.8%
Right persistent hypoglossal artery	1 (0.8%)
Basilar Artery	1 (0.8%)

*ACA* anterior cerebral artery, ICA internal carotid artery, *MCA* middle cerebral artery, *PCA* posterior cerebral artery, *PICA* posterior inferior cerebral artery, *VA* vertebral artery

**Table 2:** Properties of flow diverters that were used and evaluated in the study

Flow diverter	Manufacturer	Composition	Markers	Number of patients in this study
Derivo	Acandis GmbH, Pforzheim, Germany	Nitinol with a radiopaque platinum core for visibility	3 platinum-iridium markers on each end	23
Silk/Plus	Balt Extrusion, Montmorency, France	Nitinol	4 platinum helical strands	49
Surpass streamline	Stryker, Portage, Michigan, USA	Chromium-Cobalt, with platinum tungsten wires for visibility	Not applicable	26
Surpass evolve	Stryker, Portage, Michigan, USA	Chromium-Cobalt, with platinum tungsten wires for visibility	Not applicable	7
Pipeline/Shield	Medtronic, Dublin, Ireland	Chromium-Cobalt, with platinum tungsten wires for visibility	Not applicable	16

In a second group of patients, in vivo out-of-stent artifacts were measured. These patients were again selected based on the inclusion/exclusion criteria stated above, but a DSA examination within 6 weeks was not sought. Since stent orientation with respect to B0 affects artifact size [2], only patients with internal carotid artery (ICA) and basilar artery (BA) aneurysms that had flow diverter segments that were aligned parallel to B0 (thus perpendicular to axial slices) were chosen for the measurements. Patients with tortuous ICAs or BAs were also excluded from this group.

### MRA technique

TOF-MRA examinations were performed at 1.5 T (Achieva, Philips Healthcare, Best, Netherlands or Magnetom Symphony Tim, Siemens Healthineers, Erlangen, Germany). T1-weighted spoiled gradient echo sequences were used. The examination parameters were as follows: TR/TE = 25/6.9 ms, FA (flip angle) = 20, FOV = 230 × 195.5x112.5 mm^3^, acquisition matrix = 480 × 234, reconstruction matrix = 512 × 512, acquisition voxel = 0.48 × 0.84 × 1.5 mm^3^, reconstruction voxel = 0.45 × 0.45 × 0.75 mm^3^, pixel bandwidth = 108.5 Hz (Philips) and TR/TE = 25/7.0 ms, FA (flip angle) = 25, FOV = 230 × 195.5x112.5 mm^3^, acquisition matrix = 480 × 234, reconstruction matrix = 512 × 512, acquisition voxel = 0.48 × 0.84 × 1.5 mm^3^, reconstruction voxel = 0.45 × 0.45 × 0.75 mm^3^, and pixel bandwidth = 108.5 Hz (Siemens).

### DSA technique

Follow-up transfemoral DSA examinations included selective injections of the relevant internal carotid artery or vertebral artery (VA). Standard anteroposterior, lateral and working projections matching, as much as possible, those obtained during the endovascular treatment were acquired routinely. Occasionally, additional oblique projections and 3D rotational angiograms were obtained at the discretion of the angiographer. Nonionic iodinated contrast media with 300 mg/ml iodine concentration was used as the contrast medium for DSA. Images were obtained at 3 frames per second.

### Data collection and analysis

Patient and imaging data were examined retrospectively. The clinical and anatomical data we collected included patient age and sex, aneurysm location and the type of flow diverter used. The interval between DSA and TOF-MRA studies was also noted.

**Fig. 1:**
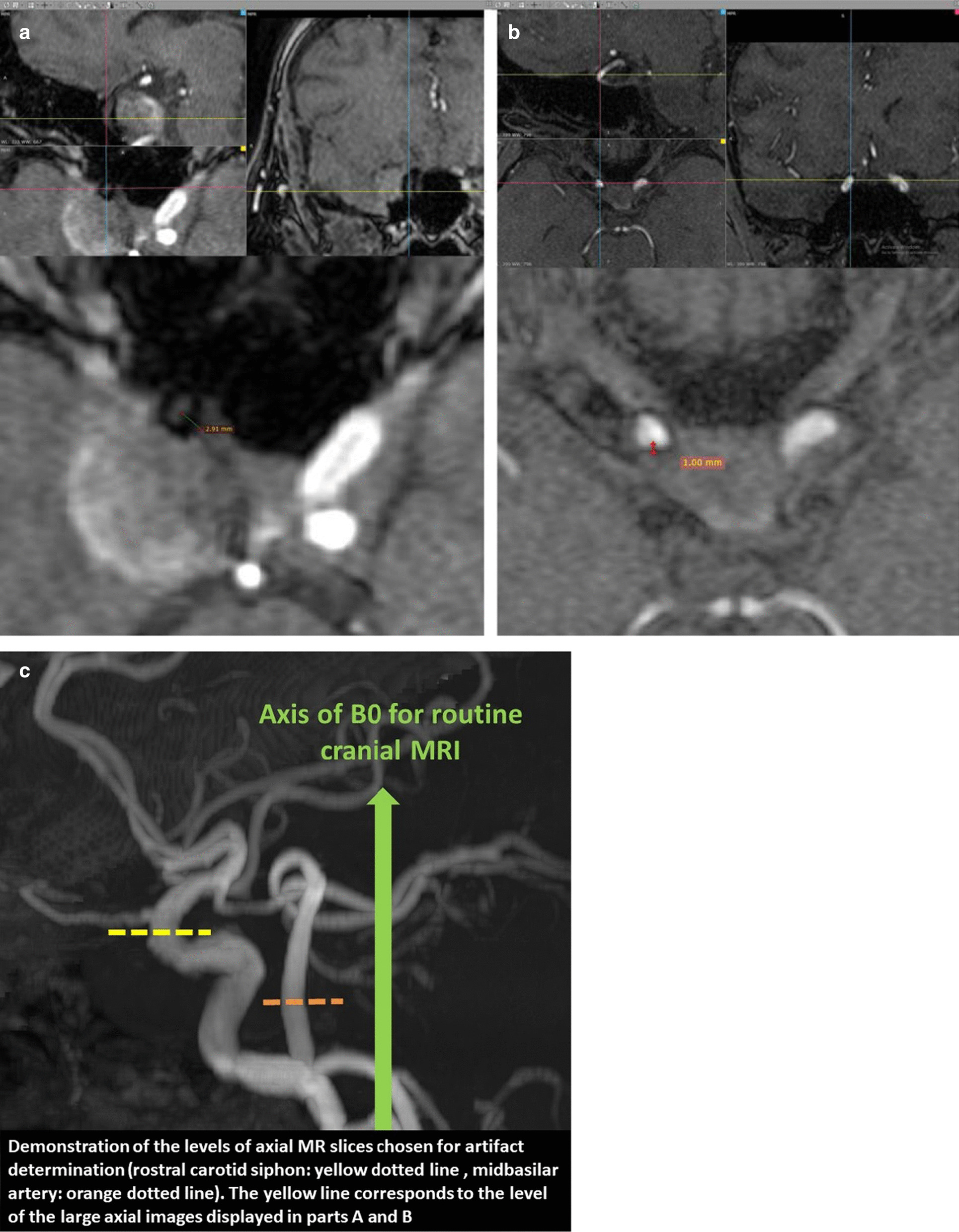
Measurement of the in vivo “out of stent” MR artifacts generated by flow diverters. **a** Patient with a chromium cobalt device. The larger axial slice in the lower part of the figure shows the measurement of the device artifact in millimeters. The point of measurement on the axial slice together with corresponding orthogonal slices (intersection of orthogonal axis, red/blue/yellow lines) are displayed in the upper part of the figure. **b** Patient with a nitinol device. As noted in the multiplanar images of both patients, the point of the measurement is at the rostral part of the carotid siphon. At this point, the flow diverter is aligned almost parallel to B0 (**c**)

MRA examinations were evaluated first. All MRA examinations were evaluated independently by 2 interventional neuroradiologists (experience: SB, 2 years in interventional neurology and 1 year in neuroradiology; BS, 3 years in interventional neurology and 5 years in neuroradiology) who were not involved in the treatment of the patients. Only the aneurysm location was provided to these readers. Then, the same readers evaluated the relevant DSA series independently. In the case of interobserver disagreement, a consensus was established before intermodality comparison. Consensus was needed in assessment of DSA images in 1 patient. Regarding TOF-MRA, consensus was established between two readers in 7 cases for aneurysm occlusion and in 11 cases for parent artery patency. For TOF-MRA, both source images and maximum intensity projection (MIP) images were analyzed on magnified views using the Radiant software (Medixant, Poland, version 4.6.5.18450). Conventional fast spin echo T1 weighted images were assessed as well to exclude T1 hyperintense thrombi in the aneurysm sac propagating as false-positive residual filling in TOF images. To evaluate the aneurysms, the Montreal scale [3] (total occlusion, neck remnant and aneurysm remnant) and then a simplified, dichotomized version of the Montreal scale consisting of either occlusion or remnant (neck remnant + aneurysm remnant) were used. For patency of the parent artery, a 3-tier scale (patent vs. stenosed vs. occluded) and a dichotomized scale consisting of either a normal artery (patent) or a pathological artery (stenosis + occlusion) were used. Additionally, to assess the in vivo “out of stent” MR artifacts generated by flow diverters, we used the same software. The orthogonal planes of MPRs were aligned perpendicular to the axis of the FD at the most rostral part of the carotid siphon (or the midbasilar artery) where the flow divert was aligned almost parallel to B0 and the size of the artifact was measured on that slice manually (Fig. 1).

### Statistical analysis

Qualitative variables are presented as numbers and percentiles. Quantitative variables are presented as means or medians with standard deviations. Sensitivity, specificity, negative predictive value (NPV), and positive predictive value (PPV) were calculated for 3D TOF-MRA with 95% CIs, accepting intra-arterial DSA as the gold standard test, both for the whole population and for each separate FD. Intermodality agreements were evaluated by calculating Cohen’s Kappa coefficient (κ). κ was interpreted as previously described [4]. Artifact sizes for different types of flow diverters were compared using the Mann–Whitney U and Kruskal–Wallis tests. Dunn’s post hoc test and Bonferroni adjustment were carried out on each pair of groups as needed. All analyses were performed with SPSS (version 25.0, Statistical Package for the Social Sciences, International Business Machines, Inc., Armonk, New York, USA).

## Results

**Fig. 2:**
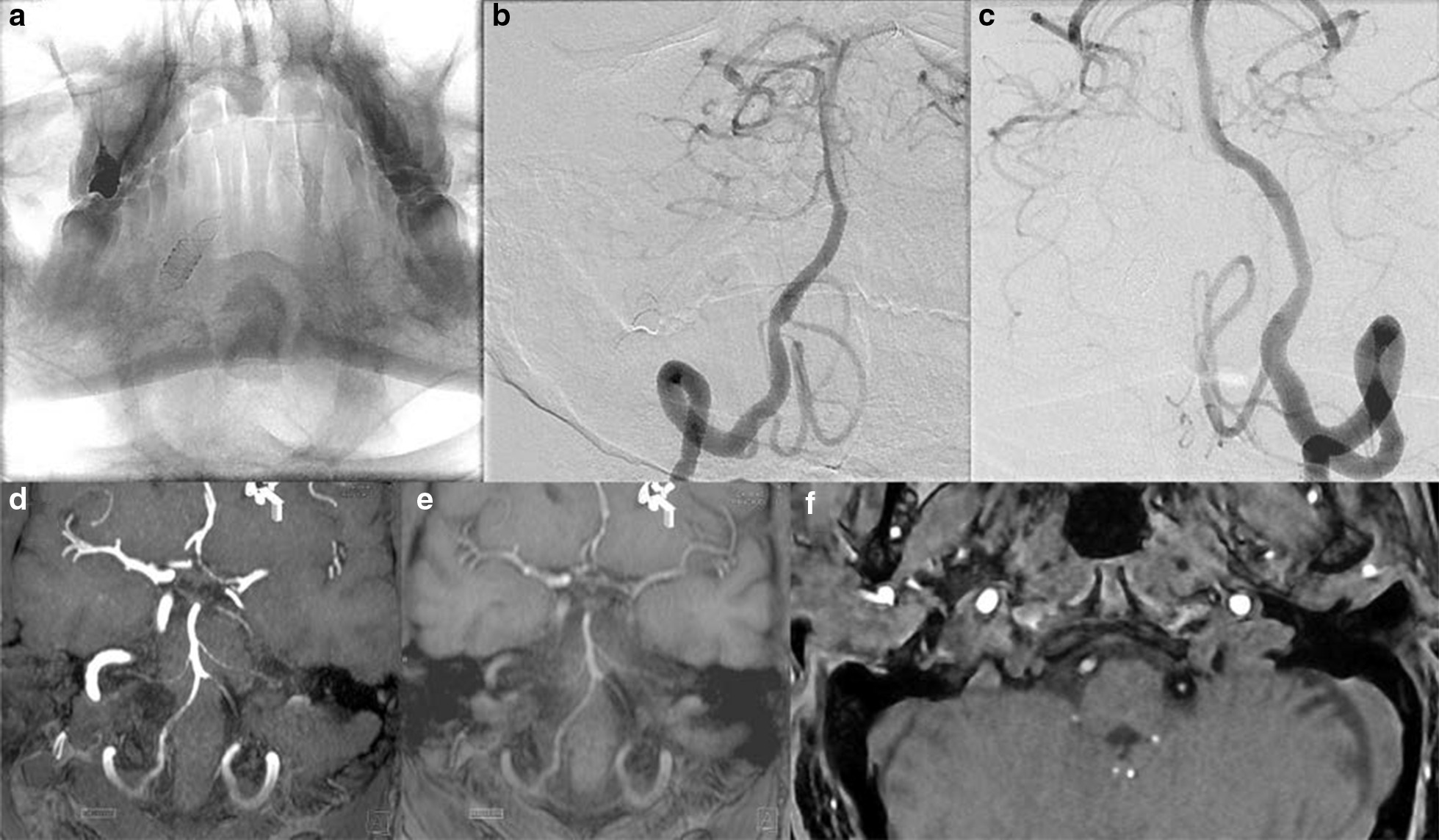
Patient in whom a Silk (nitinol device) and a Surpass Streamline (cobalt chromium device) flow diverter was placed symmetrically for unruptured dissecting V4 segment aneurysms. The native DSA image shows both devices (**a**). The subtracted DSA image from the right **b** and left **c** vertebral angiograms shows no evidence of a residual aneurysm or neointimal proliferation with significant stenosis on either side. **d** MIP reconstruction (11 mm thick) of noncontrast MRA suggests an absence of in-stent stenosis on the right and preocclusive stenosis on the left. **e** MPR reconstruction (11 mm thick) of the same image is consistent with a patent right V4 segment, whereas the left V4 segment appears occluded. **f** Note the size of the susceptibility artifact on the left side harboring the cobalt chromium device compared to that on the right side harboring the nitinol device

**Fig. 3:**
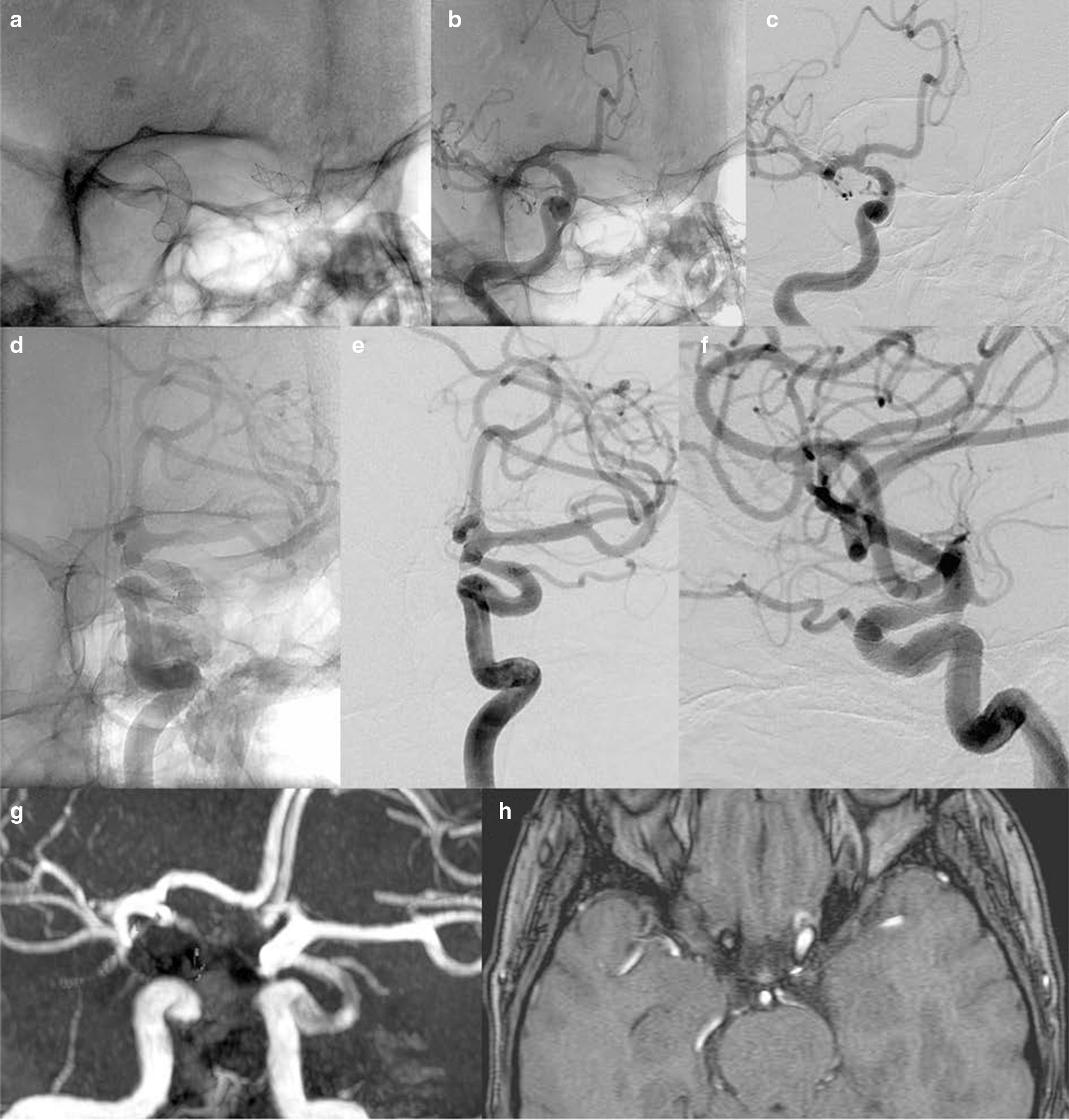
Patient with bilateral ophthalmic segment aneurysms who was treated with a Surpass (cobalt chromium) device on the right side and a Silk (nitinol) device on the left side. **a** The native image in the right oblique projection shows both devices. The native **b** and subtracted **c** images in right oblique projection of the right carotid angiogram, as well as the native and subtracted images **d**, **e** of the left carotid angiogram in the same projection and a lateral projection with caudal angulation **f** are unremarkable for residual aneurysms or parent artery stenosis. MPR reconstructions of noncontrast MRA **g** and axial source images **h** falsely suggest severe stenosis on the right but not on the left

**Fig. 4:**
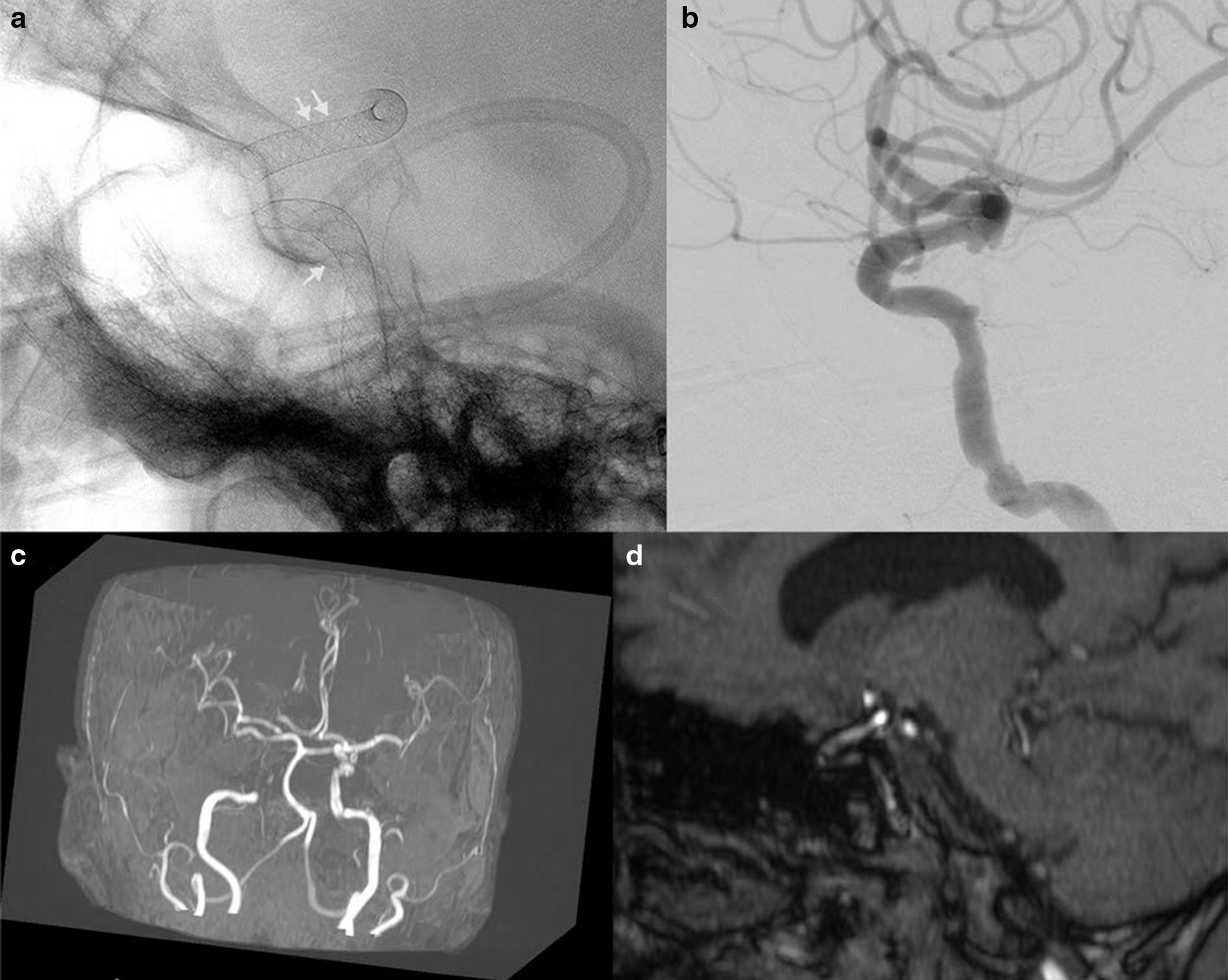
Patient with tandem petrocavernous and supraclinoid right internal carotid aneurysms who was treated with a Surpass (cobalt chromium) device for the petrocavernous aneurysm and a Pipeline (cobalt chromium) device for the supraclinoid aneurysm. The patient had 2 tandem, non-intersecting flow diverters placed for 2 tandem aneurysms and met the criteria for inclusion in group 1. The images uniquely demonstrated the difference in signal loss between two different types of adjacent flow diverters. **a** The native image shows both devices on the lateral view. The native **b** image of the right carotid angiogram shows small residual aneurysms at both locations. MPR reconstructions of noncontrast MRA **C**, **D** do not show either of the aneurysms, there is significant loss of signal in the petrocavernous segment; A loss of signal, to a lesser degree, is also evident in the supraclinoid segment

**Fig. 5:**
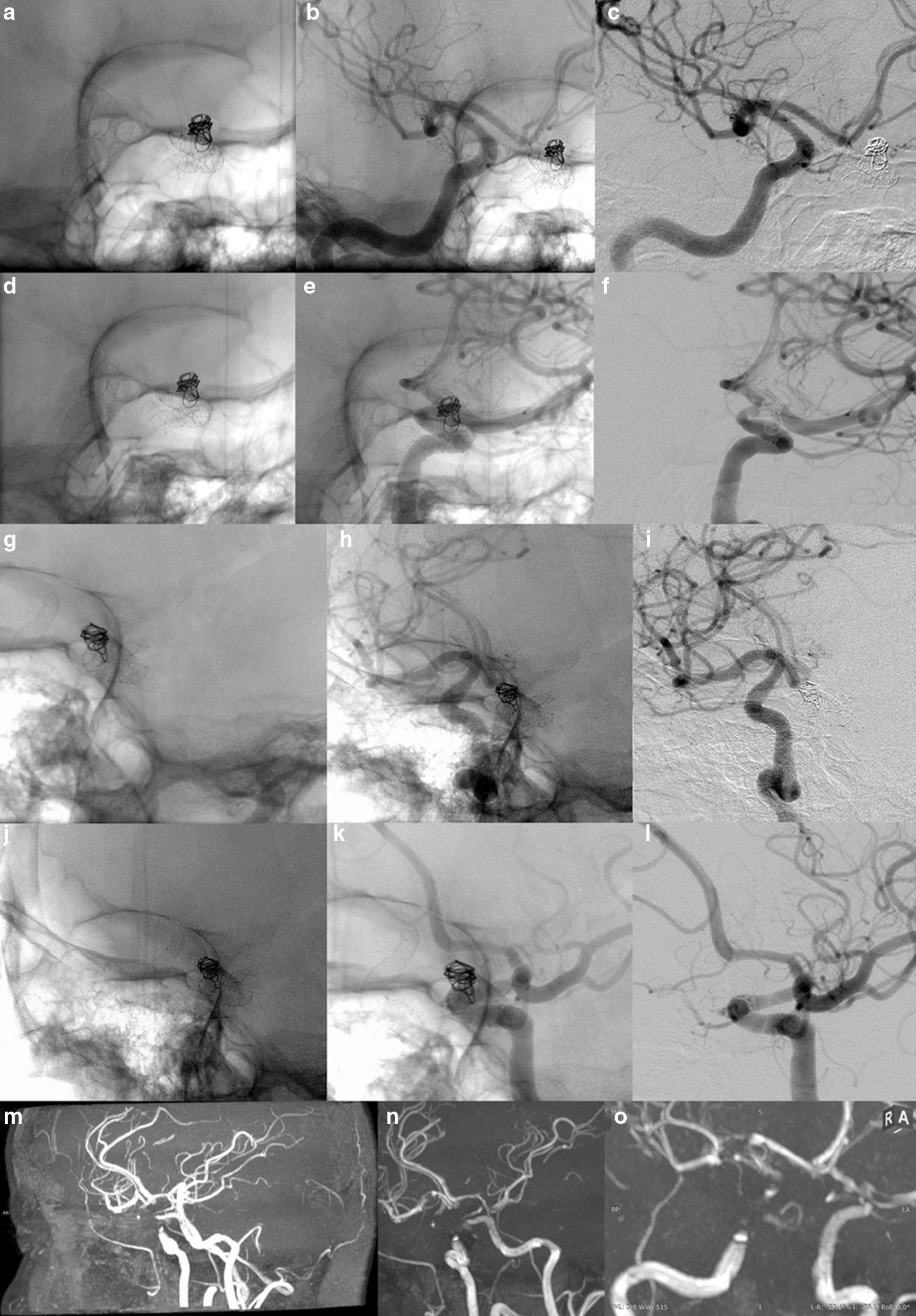
Patient with bilateral, symmetric internal carotid aneurysms who was treated with a Surpass (cobalt chromium) device on the right and a Silk (nitinol) device on the left. The aneurysm on the left was also coiled, so the patient met the exclusion criteria and was not included in the final analysis. However, the images are provided, for demonstration purposes only, to display the striking difference in signal loss, which is more significant on the right side despite the presence of coils on the contralateral side. The native images show both devices on RAO view (**a** and **d**). The native **b** image of the right carotid angiogram and the left carotid angiogram **E** along with the subtracted views **c** and **f** respectively show no evidence of a residual aneurysm or parent artery stenosis on the right or left on RAO view. The native images show both devices on LAO view **g** and **j**. Likewise, both the native views of the right **h** and left **k** carotid angiograms and also the corresponding subtracted views **I** and **l** do not reveal an aneurysm or stenosis. The MPR reconstructions of noncontrast MRA study (**m**–**o**). The left sided carotid siphon is somewhat delineated on the MIP image obtaioned without postprocessing Whereas the right siphon is not visualized (**m**). After manual removal of the posterior circulation from the MIP dataset, the LAO **n** and the RAO **o** projections that correspond to the DSA images demonstrate a substantial loss of signal on the right as compared to the left despite the presence of coils on the left which are known to cause further artifacts

**Fig. 6:**
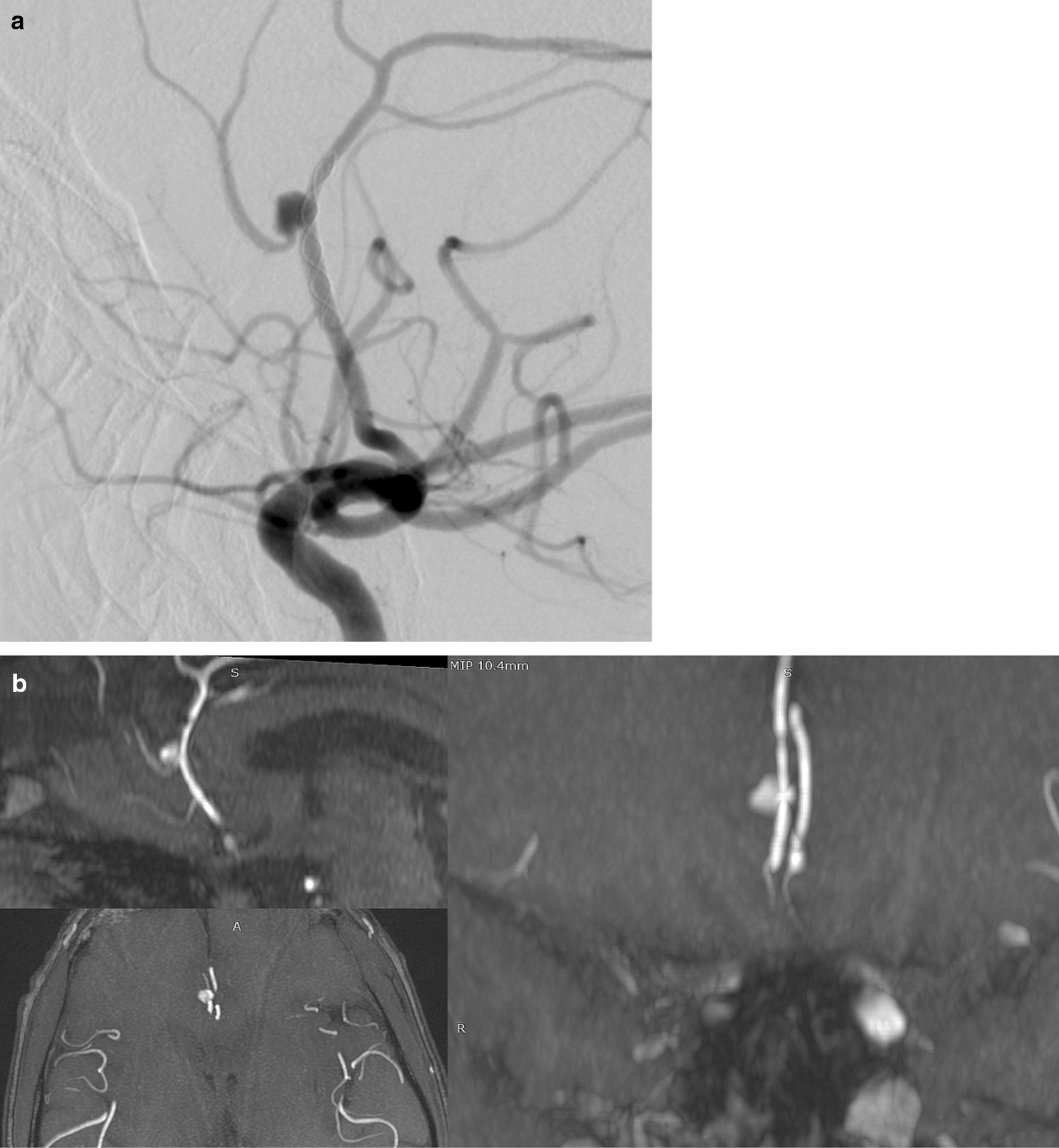
A. Distal anterior cerebral artery aneurysm, as seen on the lateral projection of internal carotid arteriogram, was treated by a nitinol device. Multiplanar MIP **b** reconstructions of TOF MRA in orthogonal planes show a residual aneurysm

A total of 111 patients harboring 121 aneurysms treated exclusively with a single flow diverter were included (Tables 1 and 2). Ten patients were treated for two aneurysms, using one flow diverter for each vascular tree. The anatomical distribution of aneurysms is shown in Table 1. Ninety-five aneurysms (94 in the ICA and 1 in the BA) were included in the artifact measurement group. Eighty-five aneurysms (59 in the ICA, 17 in the middle cerebral artery [MCA], 5 in the anterior cerebral artery [ACA], 1 in the posterior inferior cerebral artery [PICA], 1 in the vertebral artery [VA], 1 in the posterior cerebral artery [PCA], and 1 in the hypoglossal artery) were included in the TOF vs. DSA comparison group. The mean interval between the DSA and MRA was 9 ± 12 days (range 0–42 days). Examples of corresponding DSAs/MRAs are provided in Figs. 2, 3, 4, 5 and 6.

### Diagnostic accuracy of TOF-MRA vs. DSA

Thirty aneurysms were treated with chromium-cobalt-based FDs (20 Surpass Streamline (Stryker Portage, Michigan, USA), 7 Surpass Evolve (Stryker) and 3 Pipeline (Medtronic, Dublin, Ireland), and 55 aneurysms were treated with nitinol-based stents (38 Silk (Balt Extrusion, Montmorency, France) and 17 Derivo (Acandis, Pforzheim, Germany). TOF and DSA showed almost perfect agreement for the diagnosis of residual filling (κ = 0.88, *p* < 0.001) (PPV = 1.00 and specificity = 1.00 [95% CI = 0.89–1.00]) (NPV = 0.89[95% CI = 0.77–0.94] and sensitivity = 0.89 [95% CI = 0.76–0.95]). Five aneurysms that were classified as occluded according to TOF images had residual aneurysmal filling on DSA examination (Table 3). Four of these false negatives were treated with Surpass (3 Surpass Streamline, 1 Surpass Evolve), and the last one was treated with Silk. Thus, intermodality agreement was higher in nitinol stents (κ = 0.97 vs. κ = 0.74, *p* < 0.005 for both). While both materials showed excellent specificity (specificity = 1 [95% CI for chromium-cobalt = 0.72–1.00; 95% CI for nitinol = 0.84–1.00] and PPV = 1 for both), sensitivity was higher with nitinol stents (sensitivity = 0.97 [95% CI = 0.81–0.99] and NPV = 0,96[95% CI = 0.79–0.99]) than with chromium-cobalt stents (sensitivity = 0.77 [95% CI = 0.50–0.89] and NPV = 0.77[95% CI = 0.57–0.88]).

**Table 3:** Evaluation of aneurysm occlusion

	Total (n = 85)			Nitinol (n = 55)		Chromium-Cobalt (n = 30)	
	TOF-MRA		DSA	TOF-MRA	DSA	TOF-MRA	DSA
**Montreal scale**							
Total occlusion^a^		44 (52%)	39 (46%)	27 (49%)	26 (47%)	17 (57%)	13 (43%)
Neck remnant^b^		5 (6%)	5 (6%)	3 (6%)	2 (4%)	2 (6%)	3 (10%)
Aneurysm remnant^c^		36 (42%)	41 (48%)	25 (45%)	27 (49%)	11 (37%)	14 (47%)
**Two-graded simplified scale**							
Occluded^a^		44 (52%)	39 (46%)	27 (49%)	26 (47%)	17 (57%)	13 (43%)
Remnant^b+c^		41 (48%)	46 (54%)	28 (51%)	29 (53%)	13 (43%)	17 (57%)

^a^ “Total occlusion” and “occluded” represent the same group of patients with total aneurysmal occlusion per the Montreal aneurysm occlusion scale

^b+c^ Total number of “Neck remnants" (b) and "Aneurysm remnants" (c) per the Montreal aneurysm occlusion scale

TOF-MRA and DSA showed no agreement for the diagnosis of occluded or stenotic parent vessels (κ = 0.13, *p* = 0.015) (PPV = 0.15[95% CI = 0.13–0.18] and specificity = 0.44 [95% CI = 0.33–0.55]) (NPV = 1.00 and sensitivity = 1.00 [95% CI = 0.60–1.00]). Forty-three parent vessels (22 chromium-cobalt, 21 nitinol devices) that were classified as stenosed/occluded on TOF images were found to be normal on the DSA (Table 4). Kappa values were insignificant for both nitinol (κ = 0.10, *p* = 0.09) and chromium-cobalt (κ = 0.04, *p* = 0.46) stent groups. Sensitivity was perfect for both groups (sensitivity = 1.00 [95% CI for chromium-cobalt = 0.51–1.00; 95% CI for nitinol = 0.20–1.00] and NPV = 1.00). Specificity was lower in chromium-cobalt stents than in nitinol stents (specificity = 0.08 [95% CI = 0.01–0.28] vs. specificity = 0.60 [95% CI = 0.46–0.73]). Since occlusion/stenosis prevalence was low in our cohort (7 stenotic vessels and 1 occluded vessel) and distribution was not even between groups (6 pathological vessels in the chromium-cobalt group and 2 pathological vessels in the nitinol group), we have refrained from commenting on PPV.

**Table 4:** Evaluation of parent vessel patency

	Total (n = 85)		Nitinol (n = 55)		Chromium-Cobalt (n = 30)	
	TOF-MRA	DSA	TOF-MRA	DSA	TOF-MRA	DSA
**Three-graded scale**						
Total Occlusion^a^	13 (15%)	1 (1%)	1 (2%)	1 (2%)	12 (40%)	0
Stenosis^b^	38 (45%)	7 (8%)	22 (40%)	1 (2%)	16 (53%)	6 (20%)
Patent^c^	34 (40%)	77 (91%)	32 (58)	53 (96%)	2 (7%)	24 (80%)
**Two-graded Simplified Scale**						
Pathological^a+b^	51 (60%)	8 (9%)	23 (42%)	2 (4%)	28 (93%)	6 (20%)
Patent^c^	34 (40%)	77 (91%)	32 (58%)	53 (96%)	2 (7%)	24 (80%)

^a+b^ The total number of parent arteries that are either “totally occluded" (a) or "stenotic" (b)

^c^ The number of patent parent arteries

### In vivo out-of-stent artifacts generated by flow diverters on TOF-MRI

**Fig. 7:**
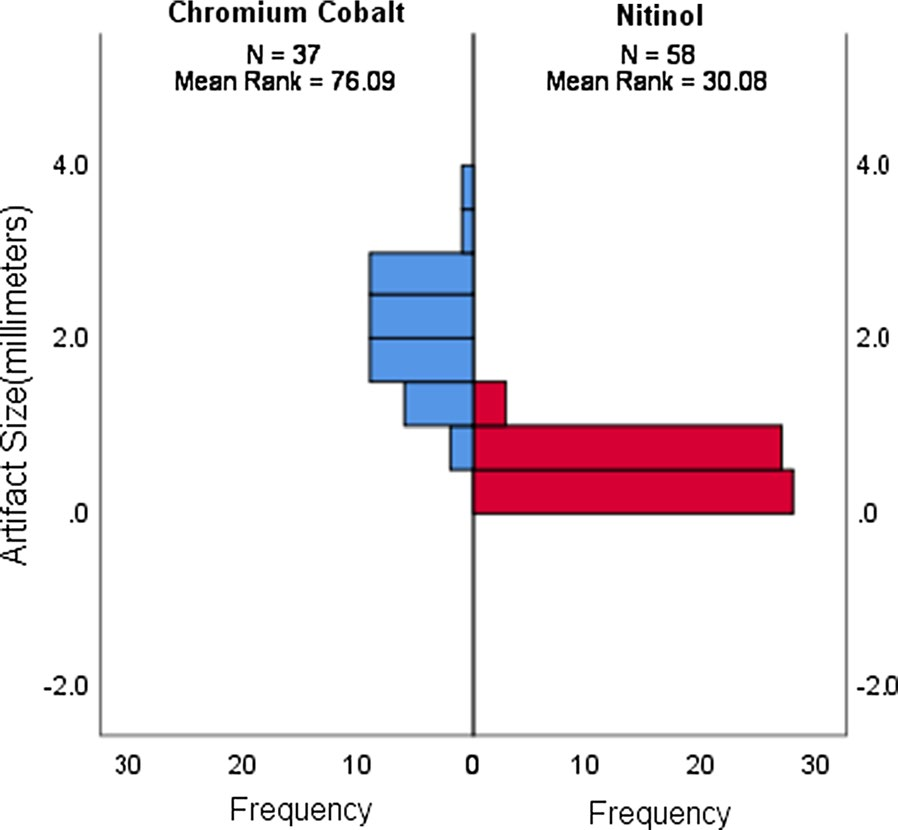
Comparison of out-of-stent artifacts generated by nitinol-based and chromium-cobalt-based flow diverters.** (**Mann–Whitney *U* test: *p* < 0.005, y axis: artifact size in millimeters, x axis: number of samples)

**Fig. 8:**
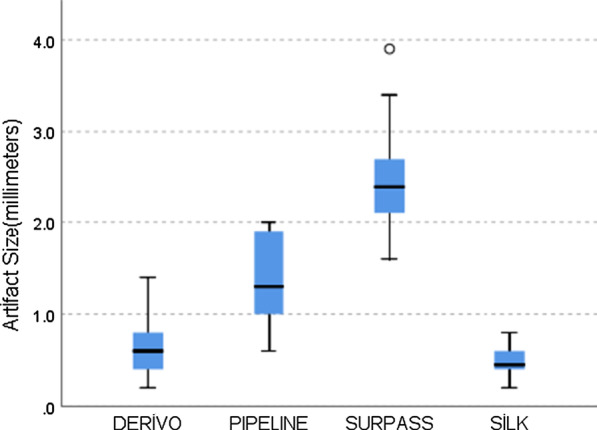
The size of device-related artifacts according to each device. (Kruskal–Wallis test, *p* < 0.005, y axis: artifact size in millimeters, x axis: type of flow diverter.) Due to the low number of Surpass Evolve patients (n = 3), Surpass Streamline and Surpass Evolve devices were grouped under a single “Surpass” group

**Fig. 9:**
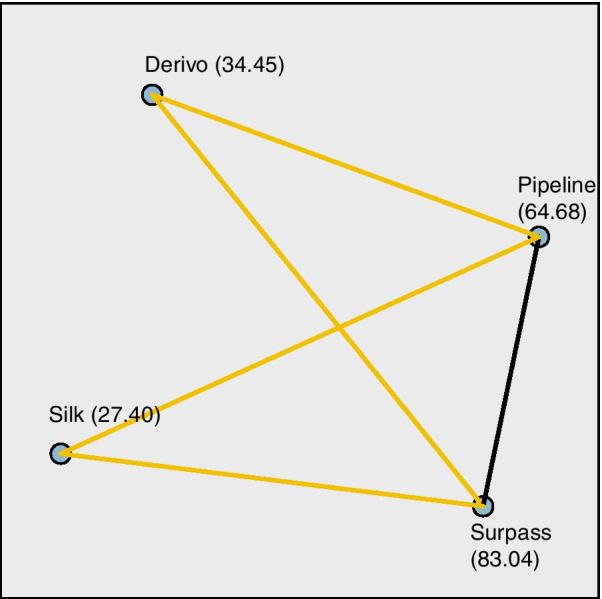
Post hoc Dunn test with Bonferroni adjustments. No difference was observed between the nitinol-based devices [Silk vs. Derivo (*p* = 1.000)]. The same is also valid for cobalt-chromium-based devices [Pipeline vs. Surpass (*p* = 0.286)]. Differences are noted between devices of different metallic compositions [Pipeline vs. Silk (adjusted *p* < 0.0005), Surpass vs. Silk (adjusted *p* < 0.0005), Pipeline vs. Derivo (adjusted p < 0.007) and Surpass vs. Derivo (adjusted *p* < 0.0005)]

The width of the signal artifact (void) was measured in millimeters for each of the 95 patients who formed the artifact measurement group. Fifty-eight aneurysms were treated with nitinol-based stents (22 Derivo and 36 Silk). Thirty-seven aneurysms were treated with chromium-cobalt-based stents (14 Pipeline, 19 Surpass Streamline and 4 Surpass Evolve). Chromium-cobalt-based stents generated larger artifacts than nitinol stents (*p* < 0.005) (Fig. 7). Based on the measurement of the size of the signal loss, the least amount of signal degradation was caused by the Silk and Derivo (0.48 ± 0.15 and 0.61 ± 0.32 mm) stents, followed by the Pipeline device (1.39 ± 0.44 mm). The Surpass devices fared the worst (2.4 ± 0.53 mm) (Fig. 8). An independent-sample Kruskal–Wallis test yielded a significant difference between at least two FD devices (*p* < 0.005). Post hoc analysis showed that there was no significant difference between stents made of the same material when compared pairwise (Derivo vs. Silk [*p* = 1.000] and Pipeline vs. Surpass [*p* = 0.286]); rather, comparisons between stents produced from different compounds caused the difference (Pipeline vs. Silk [*p* < 0.0005], Surpass vs. Silk [*p* < 0.0005], Pipeline vs. Derivo [p < 0.007] and Surpass vs. Derivo [*p* < 0.0005]) (Fig. 9).

## Discussion

Noninvasive imaging has been widely evaluated as a tool for the follow-up of aneurysms treated by flow diverters, but it has not been thoroughly validated. In a recent meta-analysis, Ahmed et al. showed that both contrast-enhanced and TOF-MRA can be used for follow-up in patients treated with flow diverters [5]. On the other hand, these authors clearly concluded that individually determined clinical factors must be taken into consideration to optimize follow-up regimens for each patient. They included aneurysm location, aneurysm size and aneurysm morphology as clinically relevant variables regarding MRA follow-up. Although these authors’ points are very well taken, we stress that the factors they cite are not modifiable variables for an individual patient. One potentially modifiable variable exists, that is, the physical properties of flow diverters, which are known to be device-specific and which were shown, in vitro, to alter aneurysm and parent artery visualization under MRI [1]. The effect of this variable on MRI imaging has not been investigated clinically.

For practical purposes, DSA results are more or less unequivocal for any type of flow diverter used. Whether this statement also holds true for MRA is yet to be demonstrated. Indeed, the data on this subject are not trivial. For example, it typically takes 6 to 12 months for the majority of flow-diverted aneurysms to occlude [6–8]. This period may be even longer for bifurcation aneurysms [9], and serial imaging is essential for these cases [10]. MRA is preferred to DSA in serial imaging due to minimal risks for complications, radiation-related risks and risks associated with repetitive administration of contrast media. More specifically, TOF-MRA has been preferred in most centers as the primary imaging follow-up modality despite some minor tradeoffs in accuracy [11], especially at high field strengths [12]. Despite the widespread use of MRA for follow-up, it remains to be shown whether different FDs are visualized under MRI differently as well. This information is important both for diagnostic neuroradiologists and for interventionalists.

Flow diverters are mainly constructed from chromium-cobalt or nitinol (Table 2). Platinum is incorporated within the device construct for visibility. However, the devices differ in strut architecture, the method of platinum incorporation, the thickness of the struts, strut angulation and the configuration or content of device markers. These differences can potentially alter the degree of artifacts they generate. These artifacts are susceptibility artifacts and shielding artifacts. The first is caused by innate magnetic susceptibility of the material used. In vitro studies [13] suggest that nitinol has a lower susceptibility than chromium-cobalt alloys. Shielding artifacts are generated by eddy currents, which are secondary to radiofrequency (RF) excitation of conductive wire loops of stents and flow diverters [1]. Braided, continuous structures create a strong shielding artifact, especially when the stent is oriented perpendicular to B0; the artifact obscures the lumen of the flow diverter and erroneously suggests vessel occlusion or stenosis [1, 2]. The importance of shielding effects in nitinol peripheral stents, which are actually less vulnerable to image degradation due to high porosity and low susceptibility, has been demonstrated [14, 15]. Furthermore, a similar shielding effect was reported for the Woven EndoBridge (WEB; Sequent Medical, Inc., Aliso Viejo, California, USA) device, which is also mainly constructed of nitinol [16].

In our study, we demonstrated that overall, TOF-MRA is fairly sensitive (sensitivity = 0.89) for residual aneurysm filling. Sensitivity was near perfect for nitinol-based stents (sensitivity = 0.97) but lower for chromium-cobalt-based stents (sensitivity = 0.77). Specificity was perfect for the whole cohort. The diagnostic accuracy of TOF-MRA for the assessment of the parent artery was rather poor, with no agreement with DSA and low specificity. Notably, nearly all parent vessels harboring chromium-cobalt stents were classified as pathological; thus, specificity was even lower for chromium-cobalt stents. With the abundance of false positives, sensitivity was perfect. Our findings in the chromium-cobalt device arm are somewhat similar to the work of Attali et al. [12], which included mostly chromium-cobalt stents (18 out of a total of 22 patients) and telescoping stents (2 of 22 patients). These authors were not able to perform a comparison of different devices in their series. Their results were apparently dominated by chromium-cobalt devices.

Out-of-stent artifacts have been measured for a variety of endovascular devices [17–20], but to our knowledge, such measurements have not been performed for flow diverters previously. The artifacts generated by flow diverters were significantly larger within the chromium-cobalt group in our cohort. This may explain the reduced sensitivity for residual aneurysms and the greatly diminished specificity for pathological parent vessels. Furthermore, the absence of differences among different stents made of the same alloys (Derivo vs. Silk and Pipeline vs. Surpass) suggests that susceptibility is a major determining factor in the accuracy of MRI in detecting residual aneurysms or parent artery stenosis.

Our results not only aid neuroradiologists in interpreting MRAs but also may have clinical implications for neurointerventionists in choosing a specific flow diverter for treatment or altering the follow-up protocol. It is worthwhile to keep the shortcomings of chromium-cobalt stents in mind when interpreting parent artery stenosis or ruling out minor residual aneurysmal filling. The interventionists, when all other things are equal, may prefer to use a nitinol-based device for blister-like aneurysms, ruptured dissecting aneurysms or recently ruptured saccular aneurysms in which the demonstration of residual minor filling is clinically important. The interventionists may also choose to have a lower threshold for obtaining a DSA during follow-up in certain patients depending on the devices used. The investigators need to be aware of potential differences among different flow diverter types under MRI when performing head-to-head outcome assessments for these devices. Finally, flow diverter vendors may consider agreeing on a standard MRA protocol in an attempt to define the MRA visibility of their devices.

## Limitations

Stent artifacts are related to the alignment of the device with respect to B0 [1]. We eliminated this effect in our artifact measurement patients by exclusively including stents placed in the ICA siphon and straight, nondolicoectatic basilar arteries and by performing measurements from the specific slice where the flow diverter lies parallel to B0. However, due to the lower number of devices in the TOF-DSA comparison group, we did not use this exclusion criterion. This resulted in an inherent limitation in our study for this arm. That is, stents placed in the M1 segment of the MCA and A1 segment of the ACA lie in a near-orthogonal plane in comparison to B0; therefore, they are expected to cause more image degradation. In addition to B0-related effects, these aforementioned vessels are prone to in-plane saturation effects [21]. The difference in vessel size and consequently the flow diverter size that matches it can be mentioned as another limitation for the TOF vs DSA arm. Prospective studies with predetermined imaging protocols that evaluate an even larger cohort of aneurysms grouped according to the size of the parent artery can overcome these limitations. Second we aimed to eliminate a recall bias by having MRA images evaluated before DSA (golden standard) images and by utilizing only the interpretations of neuroradiologists who were not involved in treatment. Yet there remained four unique aneurysm locations in this cohort (PICA, VA, PCA and persistent hypoglossal artery; with one patient in each location) and a recall bias pertaining to any of these 4 aneurysms cannot be totally excluded for the TOF vs DSA arm. Third, contrast-enhanced MRA was not included in our study because of its retrospective design. Our routine MRA follow-up involves the acquisition of contrast MRA images only when noncontrast MRA results are shown to be or are likely to be equivocal. One potential solution for this may be the use of newer techniques that were not available to us, such as “silent MRA”, which allows better assessment of the stent lumen [22]. Finally, notwithstanding the facts that our study: (1) has a fairly large number of flow-diverted aneurysms treated and followed by a single group of operators and (2) is the largest series to date focusing strictly on MRA follow-up of flow diverters, the number of aneurysms treated with the new Evolve device in the artifact measurement cohort was lowered significantly due to the selection criteria for the relevant measurement. Although we showed a difference between the artifact size measurements within the chromium-cobalt category, it was not statistically significant. Further evaluation, in larger series, can potentially disclose a significant difference among the Surpass Streamline device, Surpass Evolve device and Pipeline device, which may have been obscured by our sample size in this category.

## Conclusion

Unlike intracranial stents, which are built exclusively on a nitinol backbone, flow diverters are based on either nitinol or chromium-cobalt alloys. The architecture of these devices varies as well. Unlike the gold standard DSA, which is invulnerable to these differences, MRA is susceptible to the dissimilarities between flow diverters. Nitinol-based devices appear to be advantageous for noncontrast MRA follow-up, which remains the most frequently used imaging modality utilized for the follow-up of aneurysms treated by flow diversion.

## Data Availability

The datasets used and/or analysed during the current study are available from the corresponding author on reasonable request.

## Abbreviations

ACA: Anterior cerebral artery
BA: Basilar artery
CT: Computed tomography
CTA: Computed tomography angiography
DSA: Digital subtraction angiography
ICA: Internal carotid artery
MCA: Middle cerebral artery
MRA: Magnetic resonance angiography
NPV: Negative predictive value
PICA: Posterior inferior cerebral artery
PCA: Posterior cerebral artery
PPV: Positive predictive value
RF: Radiofrequency
TOF-MRA: Time of flight magnetic resonance angiography
VA: Vertebral artery
